# Effect of Waste Polyethylene and Wax-Based Additives on Bitumen Performance

**DOI:** 10.3390/polym13213733

**Published:** 2021-10-28

**Authors:** Luca Desidery, Michele Lanotte

**Affiliations:** 1 Department of Aerospace Engineering, Khalifa University of Science and Technology, Abu Dhabi 127788, United Arab Emirates; luca.desidery@ku.ac.ae; 2 Department of Civil Infrastructure and Environmental Engineering, Khalifa University of Science and Technology, Abu Dhabi 127788, United Arab Emirates

**Keywords:** polyethylene-modified bitumen, wax-based additives, rutting, linear viscoelastic properties

## Abstract

Over the last years, the replacement of traditional polymer modifiers with waste plastics has attracted increasing interest. The implementation of such technology would allow a drastic reduction of both production cost and landfill disposal of wastes. Among all, polyethylene-based plastics have been proved suitable for this purpose. The research activities presented in this paper aim to assess the synergistic effect of polyethylene and Fischer–Tropsch waxes on the viscoelastic properties and performance of the bitumen. In order to reduce the blending time, waxes, and polyethylene need to be added simultaneously. In fact, the presence of the waxes reduces the polarity of the bitumen matrix and increases the affinity with the polymer promoting its dispersion. Results demonstrate that the chain length of the waxes, the form of the added waste polyethylene, and the blending protocol have critical effects on the time-evolution of such properties. Short-chain waxes have a detrimental impact on the rutting resistance regardless of the blending protocol. On the contrary, long-chain waxes improve the overall behavior of the polyethylene-modified binders and, in particular, the resistance to permanent deformations.

## 1. Introduction

Over the last decades, the development of sustainable and eco-friendly technologies has become crucial to limit the environmental burden arising from ever-increasing human activities. The exponential growth of the global population and the world’s economies has led to mass production of goods and fast development of countless infrastructures and industries. Besides the huge consumption of energy and non-renewable resources, such relentless improvements are accompanied by an enormous generation of wastes and greenhouse gases that threaten our ecosystem. It has been estimated that the industrial sector accounts for approximately 20% of the total global emissions and, considering the new directives, it is required to reach adequate production processes. The paving industry is no exception and shall direct much effort to develop efficient and less polluting processes to manufacture asphalt pavements [1,2] and evaluate its carbon footprint [3,4,5].

The reduction of the working-temperature during production, laying, and compaction stages of asphalt concrete is an efficient methodology to reduce resources consumption. Nonetheless, the replacement of the traditional hot-mix asphalt (HMA) technology with an efficient warm-mix asphalt (WMA) process is not straightforward and brings some intrinsic challenges [1,6,7,8]. To date, the most investigated protocols are based on the addition of waxes, foaming agents, and various chemical additives [9,10,11]. Among those, Fischer–Tropsch (FT) waxes, unbranched saturated hydrocarbons produced synthetically from syngas, are considered the most promising due to the simultaneous beneficial effects on binders and, consequently, mixtures. In fact, while they reduce the viscosity and increase the lubricity of the bitumen at mixing and compaction temperatures, depending on the length of the wax chain, they can also generate stiffer materials at in-service conditions [12,13]. Nevertheless, efficient production and recycle processes are not sufficient to reduce long-term emissions and energy consumption if the asphalt pavements require constant maintenance and rehabilitation. High-grade asphalt concrete with specific mechanical characteristics, properly designed for specific in-service conditions, is essential to guarantee the durability of the pavements. In general, the modulation of the mechanical response of these materials involves a careful selection of proper mix design [14] and the application of suitable fillers and/or modifiers [15]. Whether the modifiers are directly mixed with the aggregates or need to be pre-blended with the bitumen depends on the available production plant and, more importantly, on the nature of the modifiers [16]. Currently, polymeric materials are the most common asphalt modifiers. They are generally pre-mixed with the bitumen to afford homogeneous mixtures and guarantee a sufficient interaction among the two components. The so-called polymer-modified binders have emerged as essential composite materials for paving manufacturing. In fact, by enhancing both the elastic recovery and the thermo-mechanical resistance of the bitumen, the addition of polymers provides high-performance asphalt concrete [15,17,18,19]. However, the higher viscosity of modified binders raises some challenges in the development of an efficient and eco-friendly technology, since high working temperatures must be avoided. The simultaneous addition of polymers and viscosity reducers is considered a suitable solution to improve the workability of modified binders, and it is currently under investigation. Nonetheless, with very few exceptions, this methodology has been only tested with the classic and most common polymeric modifiers, such as SBS [2,6,20,21,22,23,24] and crumb rubber [22,25,26,27,28,29,30,31,32,33]. Thus, intensive research is required to evaluate their effects on more innovative composites. SBS-modified bitumen (PMB) is still considered the state-of-the-art technology in pavement manufacturing, but despite the many advantages provided, styrene–butadiene–styrene thermoplastic elastomers are expensive and can raise the price of asphalt binders up to 40% [17]. The search for valuable sustainable alternatives is an ongoing process.

**Figure 1:**
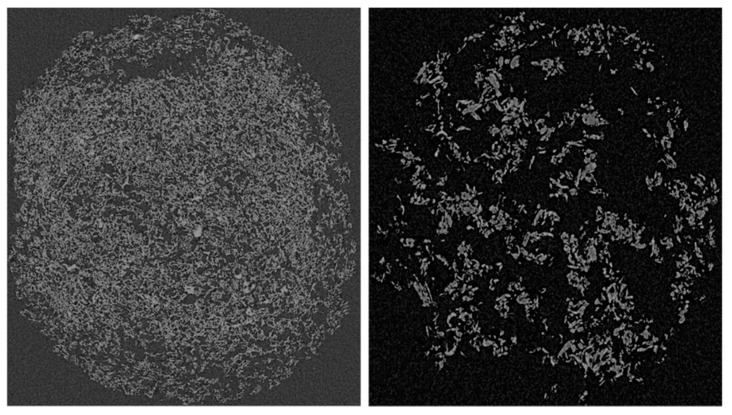
CT scan of the extracted polyethylene (**left**) and polypropylene (**right**).

Pavement engineers and researchers are devoting much effort to the application of waste and recycled materials for manufacturing high-performance concrete. Other than reducing the production costs, efficient production protocols involving such raw materials would contribute to solving the ever-increasing issue of polymeric waste disposal. Overall, plastics represent one of the greatest concerns in this regard. In fact, despite the potential capability to recycle, reuse, or upcycle several types of plastics, much of the wastes are still discarded in landfills or combusted in incinerators [34,35,36]. Such environmentally unfriendly operations drastically contribute to the pollution of water and soil, and to the emission of greenhouse and toxic gases in the atmosphere. Polyolefin-based plastics are some of the most produced plastic materials and are particularly suitable for the modification of the bitumen through traditional production protocols due to the relatively low melting point. The addition of polyethylene (PE) and polypropylene (PP) to the bitumen drastically enhances the rutting performance of asphalt binders by improving the resistance to permanent deformation and increasing its elastic recovery. The development of effective protocols to produce high-grade polyolefins-modified binders is under investigation [37]. The time-evolution of the mechanical and morphological properties during the blending process has been evaluated as a function of various independent variables, such as: form, nature, and amount of the polymer modifier. It was found that the polymer form solely affected the necessary mixing time while the final mechanical and morphological properties were related to both the nature and the amount of the modifier [37]. The mechanical characterization demonstrated that PE provided more performant asphalt binders with a higher rutting and non-loading cracking resistance. Since fluorescent microscopy was demonstrated unreliable to investigate the internal structure of this class of materials, a solvent-extraction process was ad hoc designed to recover the contained polymers without altering their structural integrity. This methodology revealed the formation of polymeric sponge-like networks within the bitumen matrixes and their tomographic analysis contributed to explaining the different rheological properties. Furthermore, the morphology of these polymeric networks was evaluated through computerized tomography (CT) scans (Figure 1) and it was observed that PE generated a much denser network of thinner and better-dispersed filaments than the one generated by PP. The structural morphology and integrity of the networks and the rheological properties of the blends are directly related to each other. The best compromise was obtained by adding 4% of polyethylene, which provided acceptable cracking and rutting resistance [37].

Building up from the above-mentioned results, the present research work aimed to investigate the effect of synthetic Fischer–Tropsch waxes on the rheological properties and performance of PE-modified binders (PEMB). The optimization of the production protocol of PEMBs containing either long- or short-chain waxes (LCW and SCW, respectively) was achieved through standardized rheological characterization methodologies. Polyethylene, both in powder and pellets, was used in this study to evaluate the contribution of the polymer form on the time-evolution of the mechanical properties of the blends.

## 2. Materials and Methods

Polyethylene-modified binders (PEMBs) containing Fischer–Tropsch waxes were prepared in the laboratory using a neat bitumen PG64-10 (NB) typically used in the country. To investigate the effect of the polymer form, a high molecular weight linear low-density polyethylene in pellets (PEPe) and powder (PEPo) were used. Two commercial Fischer–Tropsch waxes characterized by different chain lengths were evaluated in this study. The first wax consisted of a mixture of linear long-chain hydrocarbons with an average chain length of 80 carbon atoms while the second was a mixture of shorter linear waxes with an average chain length of 40 carbon atoms.

An aluminum can containing 500 g of NB was pre-heated at 180 °C in a conventional oven and then transferred into a heating mantle. The bitumen was kept under constant stirring (500 rpm) using a low-shear mixer equipped with an anchor-shaped spindle. After stabilization of the temperature, 20 g (4% by weight of NB) of polyethylene was added and the stirring rate was increased to 1000 rpm. The wax additives (1.5% by weight of NB) were either added simultaneously to the polymer or after 210 min of mixing. All blends were kept under stirring for a total of 300 min and sampled for rheological characterization after 60, 120, 210, and 300 min.

**Figure 2:**
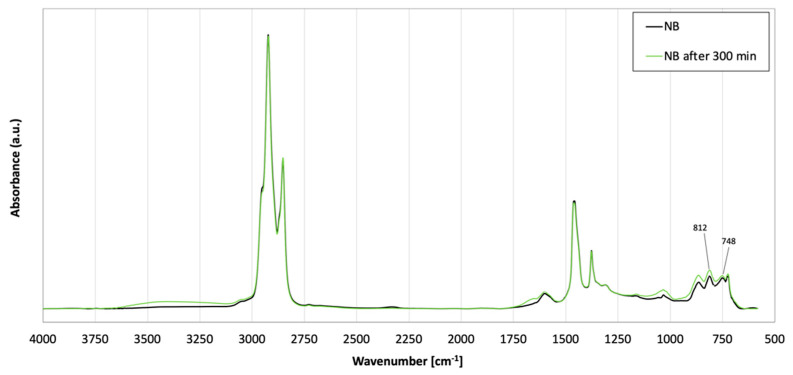
FT-IR fingerprint region of bitumen before and after 5 h mixing.

To understand the magnitude of the effects of the blending protocol (high temperature and stirring) on the chemical properties of the NB, a can of this binder was subjected to the same blending protocol and the material evaluated via Fourier Transform Infrared Spectroscopy (FT-IR). Infrared spectra were collected with a PerkinElmer Spectrum Two spectrometer (PerkinElmer, Waltham, MA, USA) in the attenuated total reflection (ATR) configuration and recorded in the frequency range comprised between 600 and 4000 cm^−1^. Results shown in Figure 2 proved that both effects, volatilization and oxidation, took place simultaneously during the high temperature blending. The appearance of the peak at 1660 cm^−1^ reflected the formation of carbonyl groups (C=O) while the more prominent peak at 1032 cm^−1^ indicated the increment of sulfoxide functionalities (S=O). Furthermore, the enhanced intensity of the peaks at 865 cm^−1^, 812 cm^−1^, and 748 cm^−1^ indicated the relative increment of the asphaltenes caused by the volatilization of lighter components. All results were in good agreement with previously reported comparisons carried out on neat bitumen before and after the rolling thin-film oven test (RTFOT) (Matest, Treviolo, Italy) aging [38]. Hence, for a better understanding of the results and fair comparison between materials, the NB was also sampled at the same sampling times mentioned above and subjected to mechanical tests.

The evaluation of linear viscoelastic properties and rutting performance of bituminous blends was conducted with an Anton Paar MCR 302 dynamic shear rheometer (DSR) (Anton Paar, Graz, Austria) equipped with parallel plate measuring systems. Frequency sweep tests were carried out using the 25- and 8-mm parallel plates at six temperatures (5, 20, 35, 55, 65, and 80 °C) in the frequency range 100–0.1 rad/s. Strain levels were defined to remain in the linear viscoelastic response region. Complex modulus (|G*|) and phase angle (δ) obtained from the DSR tests were used to evaluate the relationship between |G*| and δ in the Black Space. Furthermore, raw data were mathematically treated to build the complex modulus master curve through the application of the time-temperature superposition (TTS) principle. Such a principle allowed the visualization and further calculation of the mechanical response of asphalt binders in a wide range of temperatures and frequencies. Once a reference temperature was selected, the raw test data at different temperatures was shifted in the direction of the data obtained at the reference temperature to acquire a unique curve, namely a ‘master curve.’ In this graphical representation, datapoints at low-frequency were associated with high test temperatures and high-frequency data to low test temperatures. For this analysis, the following models were used for fitting master curve (Equation (1)) and shift factors (Equation (2)):

(1) $$\log\left(\left|G^{*}\right|\right)=\delta \;+\frac{\alpha }{1+\lambda \exp{\left(\beta +\gamma \log\left(f_{R}\right)\right)}^{\lambda }}$$

(2) $$\log\left(a_{T}(T)\right)=a_{1}\;(T^{2}-T_{ref}{}^{2})+a_{2}(T-T_{ref})\;$$

where: *δ*, *α*, *β*, *γ*, and *λ* are the coefficient of the generalized logistic sigmoidal model; *f_R_* is the reduced frequency (*f_R_* = *f*·a(*T*)); and *T_ref_* is the reference temperature set to 35 °C, in this study.

PoMBs sampled during the mixing process were subjected to multiple stress-creep recovery (MSCR) test at 64 °C, which corresponded to the high PG temperature of the NB. Following the procedure of the AASHTO M332 specification, tests were carried out by imposing two stress levels (0.1 kPa and 3.2 kPa), and for each of them, the percentage recovery (R_0.1_ and R_3.2_) and non-recoverable creep compliance (J_nr0.1_ and J_nr3.2_) were calculated. A synthetic graphical assessment of the MSCR test results was carried out by referring to the PG+ grading criteria described in AASHTO M332 specification, which identified limiting values of J_nr3.2_ for different traffic levels, namely “S” (standard), “H” (heavy), “V” (very heavy), and “E” (extremely heavy).

## 3. Results and Discussion

### 3.1. Effect of Waxes on Neat Bitumen Response

**Figure 3:**
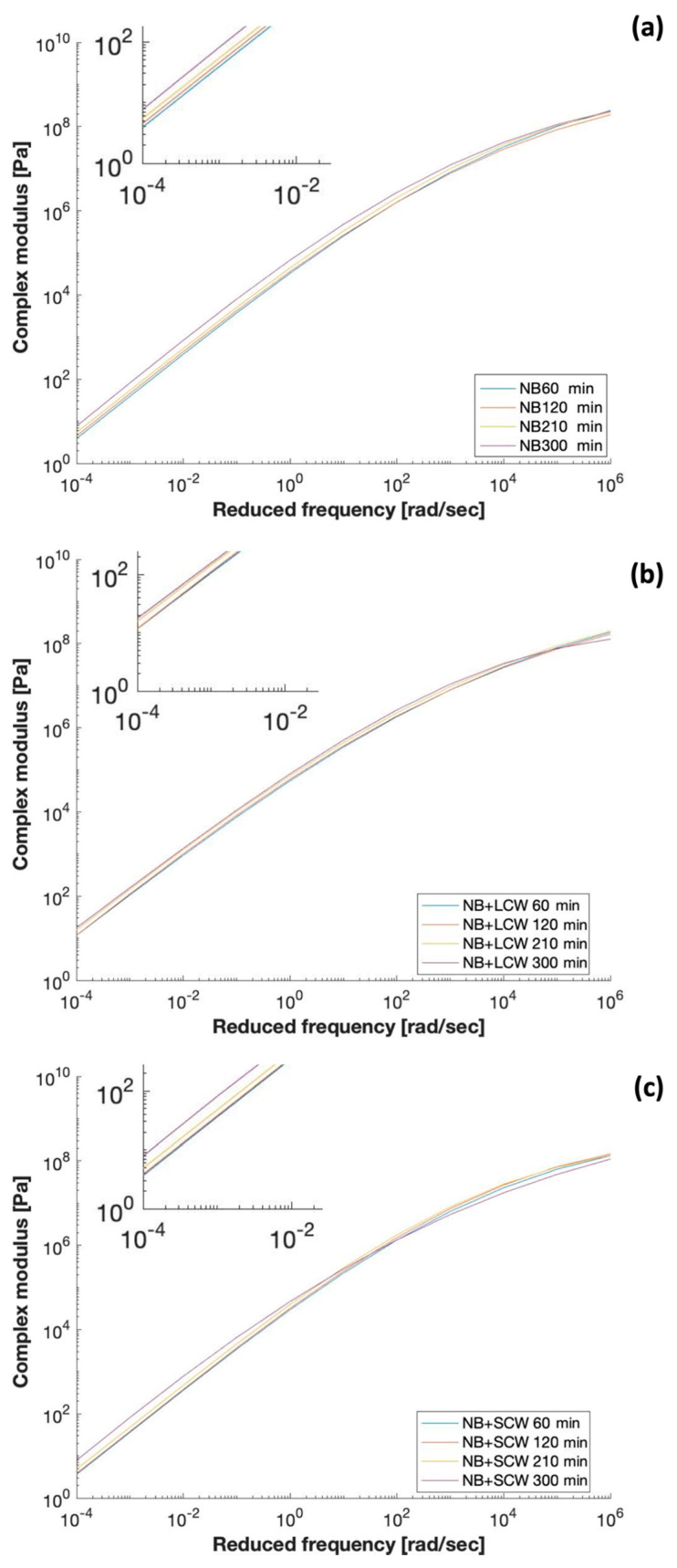
Time-evolution of linear viscoelastic properties of (**a**) NB, (**b**) NB+LCW, and (**c**) NB+SCW.

The variation of the viscoelastic response during the blending of NB and NB containing SCW and LCW is illustrated in Figure 3 by the respective master curves. The analysis of the master curves obtained on samples of NB at different blending times provide evidence, from a mechanical point of view, of what has been observed and reported in Figure 2 above. The increment of the NB complex modulus at high temperature (low frequencies) as the blending time progresses is indicative of a forced-aging process happening during blending and caused by the high-temperature. In general, aging of bitumen is one of the main factors involved in the deterioration of asphalt pavements caused by oxidation processes and volatilization of the bitumen’s light-weighted compounds [39]. Aged binders are harder and more brittle, and therefore, more prone to fracture. Since the production of polymer-modified binders requires a long mixing time at a high temperature, the aging process involved during blending cannot be neglected, as demonstrated by the experimental results.

**Figure 4:**
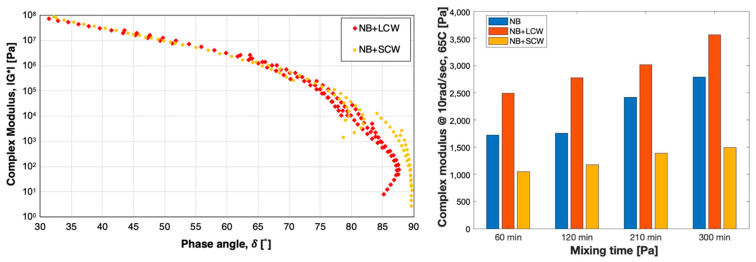
Black diagrams and complex modulus at 65 °C and 10 rad/sec after 5 h of blending.

As expected, the mechanical response of the bitumen is highly influenced not only by the presence of a wax, but also from its chain length. While the SCW tends to reduce the complex modulus of the bitumen, the LCW induces a prominent stiffening action. However, the effect of the wax is not the same at all temperatures, as it can be visualized in Figure 4 for the 300 min blending. The Black Space Diagram is often used to recognize patterns of the datasets that can be associated with the presence-bitumen modifiers. When the modifier is a wax, this data representation can be used to highlight whether the melting of the wax occurs in the measurements range of temperature and its effect on the mechanical behavior of the material. For what concerns the NB with LCW, the shape of the datasets obtained at high temperatures (high phase angles) is typical of a bitumen in which the mechanical response is still affected by the modifier. Moreover, this behavior can be recognized in NB with SCW only up to 50 °C. The datasets obtained at 65 °C and 80 °C, instead, tend asymptotically to the maximum value of phase angle (90°) typical of a fully viscous response, i.e., no interference of polymer in the mechanical response. This suggests that the melting of the SCW and the corresponding softening action of the bitumen happens at temperatures between 50 °C and 65 °C. Hence, the histogram of dynamic modulus values actually measured at 65 °C and 10 rad/s (Figure 4) was chosen for a better visualization of the magnitude of difference between |G*| values at high temperature when SCW and LCW are added to NB. Furthermore, in the presence of waxes, the induced aging effect due to the blending process is still visible even though slightly less pronounced for NB with LCW (+40%) than NB with SCW (+50%), but both lower than the NB only (+57%).

### 3.2. Effect of Waxes on Polyethylene Modified Bitumen Response

**Figure 5:**
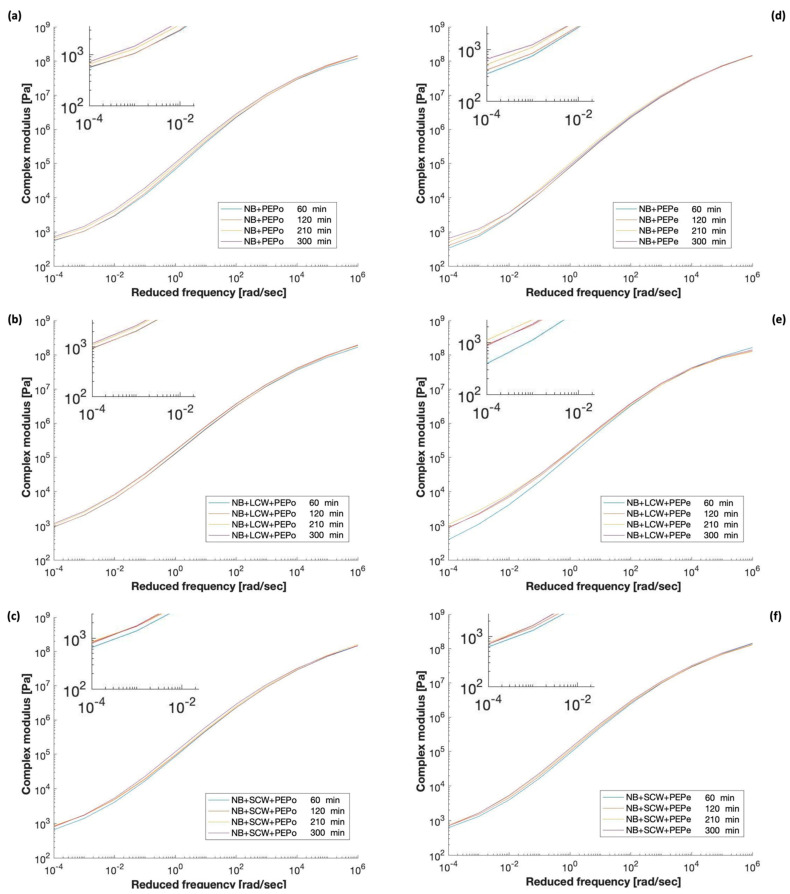
Master curves of (**a**) NB+PEPo, (**b**) NB+PEPo+LCW, (**c**) NB+PEPo+SCW, (**d**) NB+PEPe, (**e**) NB+PEPe+LCW, and (**f**) NB+PEPe+SCW.

To investigate the synergistic effect of polyethylene and Fischer–Tropsch waxes, the NB was modified with 4% of polyethylene and 1.5% of wax (by weight of the base bitumen). The amount of wax was selected following the producer’s specifications [40], whereas the amount of the PE was based on results previously obtained in our laboratories [37]. The addition of the two modifiers happened simultaneously since the presence of the wax reduces the polarity of the bitumen matrix and increases the affinity with the polymer promoting its dispersion. The effect of the mixing time on the linear-viscoelastic properties of the polyethylene-modified binders with and without waxes are represented in Figure 5.

**Figure 6:**
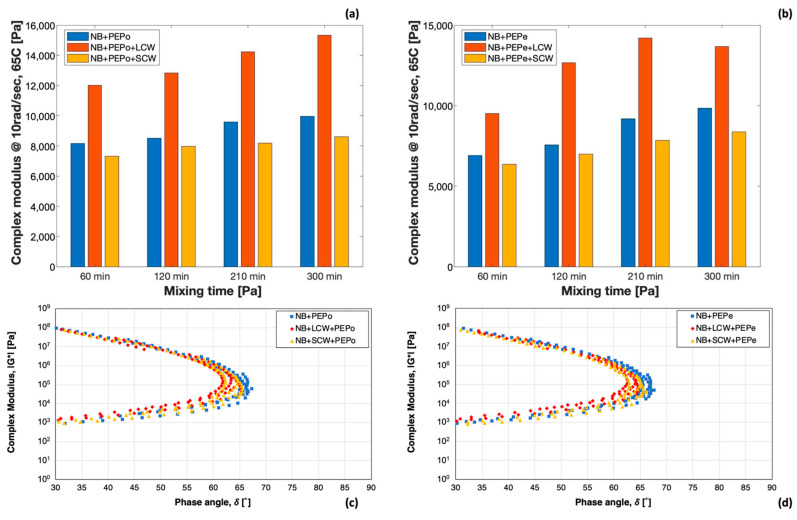
(**a**,**b**) Effect of the mixing time on the complex modulus at 65 °C and 10 rad/s and (**c**,**d**) impact of the modification on the ǀG*ǀ-δ relationships.

The tail of all curves rose progressively during the blending, indicating an increment of the modulus at high temperatures. In contrast to the results obtained with the NB or the NB containing LCW and SCW, such increment is not solely related to the forced-aging effect, but also to the formation of the sponge-like structure previously described (Figure 1). The kinetics of this process was clearly affected by the particle size of the polymer, as demonstrated by the relatively small increment of the modulus of PEPo-modified binders between 60 and 300 min. The smaller particle size of the powder facilitated the dispersion and the melting of the polymer, therefore, the formation of the polymeric network within the bitumen matrix. It is worth noticing that a similar effect can be observed for PEPe-modified bitumen containing the short-chain wax. The explanation of this event is most likely twofold and related to the impact of SCW on both fluidity and stiffness of the bitumen. The presence of SCW drastically reduced the viscosity of the binder, accelerating the dispersion of the polymer and the interaction with the bitumen. On the other hand, the increment of the modulus associated with the formation of a well-structured polymeric framework cannot compensate the softening action induced by the waxy additive. In fact, as described previously in the case of the NB only, the SCW reduced the stiffness of all plastic modified binders, regardless of the polyethylene form or blending time. On the contrary, and again as observed for the NB only, the LCW increases the modulus at high temperature and generated stiffer materials. A better visualization of the above-mentioned phenomena and their relative magnitude is provided by the dynamic modulus at 65 °C and 10 rad/s (Figure 6). 

Such graphs highlight the profound impact of the long-chain wax on the high-temperature modulus of the materials. The significant enhancement of the stiffness is clearly more predominant in respect to the softening action caused by the short-chain wax. Additionally, the histograms allow an easy comparison of the different effects induced by the two forms of polyethylene. Each PEMB produced with pelletized plastic presented a higher modulus compared to the corresponding blend produced by powdered plastic. 

Figure 6c,d show the Black Space Diagrams of PEPe- and PEPo-modified binders with and without waxes at the end of the blending process (300 min). While the increment of the temperature lead to a progressive decrement of the complex modulus, the phase angle reached a maximum value at intermediate temperatures and subsequently decreases. Although this response is typical of high polymer modified, the Black Diagram of the binders tested follows a peculiar path related to the mechanical response of the polymer and, in this case, to the polymeric network within the bitumen matrix. For temperatures higher than 50 °C, bitumen increasingly softened and the response of the polyethylene network to the stress applied by the rheometer lead the overall response of the blend. Both waxes slightly increased the degree of elastic response of the blends. However, the magnitude of their effect was affected by the chain length.

### 3.3. High-Temperature Performance

**Figure 7:**
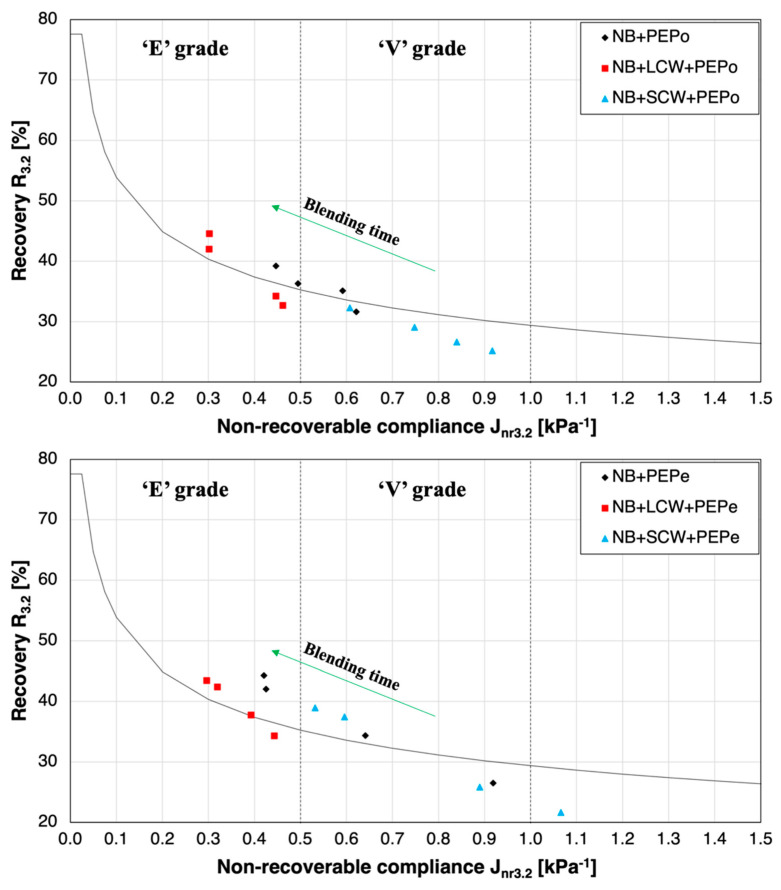
Effect of the mixing time on the rutting performance of all blends.

The time-evolution of the rutting performance of PEMBs with and without waxes was evaluated via MSCR analysis and confirmed the indications obtained from the linear viscoelastic analysis. The resistance to permanent deformations of PEPe- and PEPo-modified binders, expressed in terms of non-recovery compliance (J_nr, 3.2_) and percentage of elastic recovery (%R_3.2_), is reported in Figure 7. Both the LCW and the SCW exerted a remarkable impact on the final performance of the blends and the specific effects were related to the length of the molecular chains of the waxes, as previously observed. The presence of LCW allowed the retention of the classification grade for extremely heavy traffic (“E”) regardless of the polyethylene form added to the NB. Moreover, if compared to the bitumen modified with polyethylene only, the presence of LCW also improved the rutting resistance of the final asphalt binder. It is also worth noticing that the impact on the performance of the two blends is slightly different. On the one side, the LCW in the PEPe-modified bitumen with LCW reduced the non-recovery compliance without having a significant effect on the elastic recovery when compared to the bitumen with PEPe only. On the other side, when the LCW was added with polyethylene in the powdered form, it also led to the increase of elastic recovery. Additionally, the MSCR results revealed how the addition of the LCW strongly affected the progression of the performance during the blending time of the bitumen modified with PEPe. The data points corresponding to different blending times were much more clustered and the overcome of the elastic recovery pass-fail threshold took place after only 120 min of blending. Such event was not evident in the case of the PEPo-modified binder since the dispersion and the melting of extremely fine particles resulted in fast processes, even in the absence of wax additives. The addition of SCW, instead, is clearly detrimental in terms of the rutting performance of PEMBs. Regardless of the plastic form, the MSCR datasets fully remained in the ‘V’ bumping-grade identified by the J_nr,3.2_ limit (0.5 < J_nr,3.2_ < 1.0). Furthermore, it is worth observing that the data points associated with the PEPo-modified binder did not even overcome the pass-fail limit. Hence, the performance of the material was not satisfactory.

## 4. Conclusions

The research work herein presented aimed to evaluate the impact of synthetic Fischer–Tropsch waxes on the rheological properties and performance of waste polyethylene-modified bitumen. A comparative investigation was carried out by testing bituminous blends containing long- and short-chain waxes and waste polyethylene in both powder and pellet form. The main findings can be summarized as follows:

- The mechanical response of NB and wax-modified binders are influenced by a forced aging effect caused by the volatilization of lightweight components and oxidation reactions triggered by the high-temperature treatment to which the binders are subjected to during the blending protocol. For comparative purposes, these effects are not negligible since the modulus of the binders, particularly at high temperatures, progressively increases during the mixing procedure.
- The addition of linear Fisher-Tropsch waxes to base asphalt has a drastic impact on the rheological properties. Within the range of temperatures investigated, the specific contributions to the overall behavior can be correlated to the wax’s chain length and opposite effects originate from the addition of short-chain and long-chain waxes. In fact, when LCW is added, the mechanical response is similar to those poorly polymer-modified binders in the entire range of temperature evaluated. On the contrary, SCW-modified bitumen experiences a sudden change of behavior for temperatures above 50 °C, when the SCW starts melting and softening the bitumen.
- The modulus at high temperature of all PEMBs, regardless of the form of the waste polyethylene added, increases with blending time too. However, in contrast to the NB only and binders containing sole wax, such an event is not exclusively related to the forced aging effect, but also to the formation of a polymeric sponge-like network within the bitumen matrix.
- Both linear viscoelastic evaluation and rutting characterization through MSCR tests indicated that the use of SCW is discouraged for those environments characterized by high air temperatures and/or high traffic volumes. If rutting is expected to be the leading distress, the use of LCW is instead suggested.
- Despite the polymer form, the addition of SCW provides a detrimental impact on the rheological properties and performance of PEMBs. On the contrary, the presence of the LCW enhances both the complex modulus and the resistance to permanent deformation.

## Data Availability

The data presented in this study are available on request from the corresponding author.
